# Repeatability and Reproducibility of Noninvasive Keratograph 5M Measurements in Patients with Dry Eye Disease

**DOI:** 10.1155/2016/8013621

**Published:** 2016-04-12

**Authors:** Lei Tian, Jing-hao Qu, Xiao-yu zhang, Xu-guang Sun

**Affiliations:** Beijing Institute of Ophthalmology, Beijing Tongren Eye Center, Beijing Tongren Hospital, Capital Medical University and Beijing Ophthalmology & Visual Sciences Key Laboratory, Beijing 100730, China

## Abstract

*Purpose*. To determine the intraexaminer repeatability and interexaminer reproducibility of tear meniscus height (TMH) and noninvasive Keratograph tear breakup time (NIKBUT) measurements obtained with the Keratograph 5M (K5M) in a sample of healthy and dry eye populations.* Methods*. Forty-two patients with dry eye disease (DED group) and 42 healthy subjects (healthy group) were recruited in this prospective study. In all subjects, each eye received 3 consecutive measurements using the K5M for the TMH and NIKBUTs (NIKBUT-first and NIKBUT-average). And then a different examiner repeated the measurements. The repeatability and reproducibility of measurements were assessed by the coefficient of variation (CV) and intraclass correlation coefficient (ICC).* Results*. The repeatability and reproducibility of TMH and NIKBUTs were good in both DED and healthy groups (CV% ≤ 26.1% and ICC ≥ 0.75 for all measurements). Patients with DED showed better intraexaminer repeatability for NIKBUTs, but worse for TMH than healthy subjects. Average TMH, NIKBUT-first, and NIKBUT-average were significantly lower in DED group than in healthy group (all *P* values < 0.05).* Conclusions*. Measurements of TMH and NIKBUTs obtained with the K5M may provide a simple, noninvasive screening test for dry eye with acceptable repeatability and reproducibility. The NIKBUTs were more reliable, but TMH was less reliable in patients with DED.

## 1. Introduction

Dry eye disease (DED) is a chronic, multifactorial disease of the tears and ocular surface, which is caused by either decreased tear production or increased tear film evaporation [1]. DED is one of the most common ocular disorders, with symptoms affecting 5–30% of the population worldwide; however in many cases it is underdiagnosed and left undertreated [2]. The cornea is the transparent front part of the eye and the tear film ensures a smooth refracting surface and prevents microbial invasion [3]. As a result, the instability of a disrupted tear film over the irregular surface of a dry eye is thought to affect the quality of vision [4]. Many attempts have been made to define the characteristics of dry eye; however, no “gold standard” exists till now. Traditionally, common objective clinical measures assessing the tear film and diagnosing DED are known as the fluorescein tear breakup time (FBUT) and Schirmer test [5, 6]. But the traditional objective tests are often limited by their invasiveness and low test repeatability and reproducibility [7, 8].

The tear meniscus refers to the tears lying. It has been estimated that 75–90% of tear volume is accounted for by the tear meniscus [9]. Some previous studies reported that a positive correlation between the tear meniscus height (TMH) and Schirmer test value has been found [10, 11]. So the TMH can be considered as a noninvasive test for the quantitative of tears [12].

Recent advanced Placido topograph, the Keratograph 5M (K5M; Oculus Optikgeräte GmbH, Wetzlar, Germany), has additional imaging modalities designed to noninvasively measure TMH and noninvasive Keratograph tear breakup time (NIKBUT) [13–15]. And it has been used in the evaluation of tear film and diagnosis of DED [12]. In this study, the intraexaminer repeatability and interexaminer reproducibility of the measurements for TMH and NIKBUTs were evaluated, and their results in the DED patients and the healthy population were compared.

## 2. Methods

### 2.1. Subject Recruitment

This prospective study involved 84 eyes of 84 subjects: 42 eyes with DED not associated with Sjögren's syndrome (DED group) and 42 healthy control eyes (healthy group). In patients who were diagnosed with DED in only one eye, that eye was selected for measurement. For participants with DED in both eyes and for healthy subjects, right eye was selected for measurement and statistical analysis. The diagnosis of dry eye was made according to the consensus of DED in China (2013): (1) at least 1 of 6 symptoms: dryness, burning, sandiness, tiredness, discomfort, and blurred vision with FBUT ≤5 s or a nonanesthesia Schirmer Ι test value ≤5 mm/5 min; (2) at least 1 of 6 symptoms: dryness, burning, sandiness, tiredness, discomfort, and blurred vision with 5 s < FBUT ≤ 10 s or 5 mm/5 min < nonanesthesia Schirmer Ι test ≤ 10 mm/5 min, accompanied by corneal fluorescein staining score. Exclusion criteria for both groups were as follows: age <18 years, subject unable to complete the questionnaire or understand the procedures, the presence of ocular or systemic disease or the use of topical or systemic medications that may affect the cornea and the ocular surface (except the use of nonpreserved tear substitutes in the DED group), and previous eye surgery or contact lens wore in the past 24 hr.

Data were collected from July to December 2015 in Beijing Tongren Hospital, Beijing, China. All participants signed an informed consent form in accordance with the tenets of the Declaration of Helsinki and this study was approved by the institutional review board of Beijing Tongren Hospital, Beijing, China.

### 2.2. Ocular Examinations

Each patient was asked to complete the Ocular Surface Disease Index (OSDI) questionnaire (range: 0–100). In all eyes, ophthalmic examination was performed in the same order as follows: firstly, TMH measurement and then NIKBUTs measurement with Keratograph 5M, FBUT assessment, corneal and conjunctival fluorescein staining, nonanesthetized Schirmer Ι test, and corneal sensation measured with the Cochet-Bonnet esthesiometer (Luneau, Prunay-Le-Gillon, France).

### 2.3. Keratograph 5M Measurement

**Figure 1:**
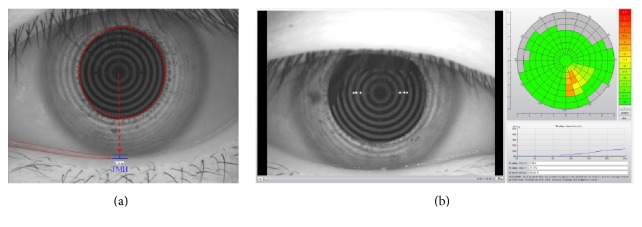
The representative outputs of TMH and NIKBUT. (a) The TMH was measured perpendicular to the lid margin at the central point relative to the pupil center. (b) NIKBUT result map included the color-coded map, breakup characteristics map, and first and average breakup time and classification.

All subjects underwent imaging with the K5M equipped with a modified tear film scanning function. In each subject, inferior TMH images were captured and measured perpendicular to the lid margin at the central point relative to the pupil center using an integrated ruler. The principle and technique for NIKBUT measurements have been described previously [12, 16]. NIKBUT was measured as the time in seconds between the last complete blink and the first perturbation of placid rings projected onto the surface of the cornea, which the device automatically detects. K5M generated two measures for NIKBUT: the time at the first breakup of tear film occurs (NIKBUT-first) and the average time of all breakup incidents (NIKBUT-average). The representative outputs for TMH and NIKBUT were shown in Figure 1.

### 2.4. Fluorescein Tear Film Breakup Time and Corneal Staining Score

Fluorescein dye was used to assess corneal staining and FBUT. A sterile fluorescein strip moistened with ocular irrigation solution was applied to the inferior fornix. Two or three minutes later, the subjects were requested to blink several times to ensure adequate mixing of the dye and then keep their eyes open. FBUT was examined under standard illumination using a slit-lamp microscope with a cobalt-blue filter, and the time was recorded with a stopwatch. FBUT is the time interval between the last blink and the appearance of the first random dry spot on the corneal surface. The average of three consecutive FBUT values was calculated. Corneal and conjunctival staining was evaluated under a yellow filter using the Oxford scale and after instillation of fluorescein.

### 2.5. Schirmer I Test

Schirmer Ι test was a useful assessment of aqueous tear production. The inferior conjunctival fornix was dried with a cotton stick. One minute later, a standard 5 × 40 mm Schirmer test strip was placed over the junction of the middle and outer third of inferior lid. The patients are instructed to keep their eyes closed during the test. The test lasted 5 minutes, and the amount of wetting was recorded.

### 2.6. Repeatability and Reproducibility of the TMH and NIBUT Measurements

To measure the intraexaminer repeatability, the TMH and NIBUT were calculated using 3 consecutive measurements by the same masked clinician. To measure interexaminer reproducibility, the participants were tested by 2 independent and well-trained clinicians in random order, and the agreement between them was analyzed. The participants were given a 10-minute pause between each measurement. All the evaluators were masked to the subjects' clinical and demographic details. All the measurements were taken between 10:00 a.m. and 16:00 p.m. in one day and in a dimly lit room where the temperature (20–25°C) and humidity (30–40%) were controlled.

### 2.7. Statistical Analysis

Two software programs, SPSS version 17.0 (SPSS, Inc., Chicago, IL, USA) and MedCalc 13.0 (MedCalc Software, Ostend, Belgium), were used to conduct the statistical analyses. Data were test for normality using the Kolmogorov-Smirnov test, which were here provided as the mean and standard deviation (SD). Differences between groups (DED and healthy) were evaluated using the Welch modified Student two-sample *t*-test and the Wilcoxon rank-sum test. A *χ* ^2^ test was performed for gender distribution. To assess intraexaminer repeatability and interexaminer reproducibility, the within-subject SD (*S*_*w*_), precision (1.96*S*_*w*_), repeatability (2.77*S*_*w*_), and coefficient of variation (CV) were calculated from the 3 consecutive K5M measurements [17]. The intraclass correlation coefficient (ICC) was also applied for the interexaminer repeatability (ICC ≥ 0.75 indicated good reliability) [18]. All *P* values were 2-sided and considered as statistically significant when <0.05.

## 3. Results

### 3.1. Demographics

A total of 42 dry eye patients and 42 healthy subjects were recruited for the study.  Table 1 showed that there was no significant difference in age and gender distribution between the two groups. The OSDI and Oxford scale values were significantly less, while FBUT, Schirmer test, and corneal sensation values were significantly more for the DED group than for the healthy group. The TMH and NIKBUTs values were also significantly lower in the DED group.

### 3.2. Intraexaminer Repeatability and Interexaminer Reproducibility

Table 2 showed the mean values, precision, repeatability, CV%, ICC, and 95% confidence interval of the TMH, NIKBUT-first, and NIKBUT-average for the 3 consecutive repeated measurements in DED and healthy groups. The CV% values were within 26.1%, and the ICCs were more than 0.75 for all parameters. Thus, the intraexaminer repeatability of TMH and NIKBUTs measurements by the K5M was good.

Table 2 also showed the mean values, precision, repeatability, and CV% of the TMH, NIKBUT-first, and NIKBUT-average for the interexaminer reproducibility. The CV% values were within 21.85%, and the precision values were within 3.94 and the repeatability values were within 5.14. These also indicated good interexaminer reproducibility.

## 4. Discussion

The tear film is essential for maintaining the health of the ocular surface and also it is an important optical element, which ensures a smooth refracting surface [19]. It forms a complex and stable system in ocular surface. As a result, the instability of a disrupted tear film may compromise ocular health and lead to dry eye. In clinical practice, FBUT is the most widely performed examination to aid in assessing the tear film stability. Although FBUT measurement using fluorescein dye is a minimally invasive technique, fluorescein instillation can destabilize the tear film [7]. The Schirmer test, on the other hand, is the most commonly used test to measure tear production, which is an indispensable component of examination in patients with DED. But it has been suggested to have low reproducibility, with wide variations occurring between subjects and on different days/visits, and the reliability of the test can be affected by environmental conditions, for example, temperature and humidity [8, 20]. Non- or minimally invasive dry eye tests have the major advantage without significantly inducing reflex tearing, which can subsequently affect results following the invasive procedure. These types of noninvasive techniques, such as K5M, have the potential to represent the “true” state of the ocular surface [5]. In the current study, the TMH and NIKBUTs were measured using K5M in patients with DED and healthy subjects. To the best of our knowledge, this is the first study to compare the repeatability and reproducibility of TMH and NIKBUTs measured by K5M in patients with DED. The results of this study reveal the good repeatability and reproducibility of TMH and NIKBUTs measurements. Patients with DED exhibited lower TMH and shorter NIKBUTs than healthy subjects.

“Repeatability” is defined as the variability in repeated measures by one examiner without changing all other factors. “Reproducibility” refers to the variability in repeated measures when factors are varied [21]. The importance of longitudinal observation of clinical findings in diagnosis and treatment emphasizes the importance of repeatability of its measurements and assesses the reproducibility of its readings with different examiners, when a new instrument is used in clinical practice. Previous repeatability studies of Oculus Keratograph systems have been conducted predominantly on healthy subjects [22], but the repeatability and reproducibility measures in patients with DED were reported rarely; therefore, understanding of the performance of K5M test in a dry eye sample is largely unknown.

Previous studies have evaluated repeatability of NIKBUTs in healthy subjects and have reported results ranging from good reliability [16] to poor reliability [22]. Consistent with the 95% limits of agreement, the ICCs for the NIKBUTs were good in the current study. This study found that the NIKBUTs were more reliable tests in DED group than in healthy group in producing less varied results and more repeatability. Differences in the measurements can be attributed not only to the instrument and operator but also to changes that occur in the eye. According to reduced corneal sensitivity reported in dry eye patient populations, the influence of reflex tearing was less in DED eyes than in healthy eyes [12]. These may explain, in part, why the reliabilities of NIKBUTs were higher in DED group in this study. The relationship between tear function or stability and corneal sensitivity in DED is of interest and should be clarified in future studies. Although the corneal epithelial abnormalities, which presented as corneal staining, may influence the result of repeatability and reproducibility measurements, there were a few eyes showing staining in the DED group, so they can be ignored.

The reliability for measuring TMH already had been established in healthy population with a good intraexaminer repeatability (CV% = 0.16% and ICC = 0.83, resp.) [14], but until now there is no data in patients with DED. Our results showed that the repeatability and reproducibility of TMH reached a good level in DED group, but the TMH was less reliable than healthy subjects. K5M also has its shortcomings: the eyelid margin or the upper margin of the lower meniscus cannot be delineated automatically and the image obtained with the K5M was poor, which made it difficult to correctly delineate the tear meniscus. All of these might compromise the repeatability and reproducibility of its measures.

On the basis of the measurement repeatability and reproducibility, the NIKBUT-first and NIKBUT-average in DED group were significantly shorter than those in healthy group in this study (Table 1). Koh et al. [23] report NIKBUT-first values of 9.71 ± 6.68 s for the healthy eyes and 4.59 ± 1.25 s for the dry eyes. Our results of the NIKBUT-first values were consistent with Koh et al.'s finding, whereas the NIKBUT-first values obtained in Hong et al.'s study [16] (4.3 ± 0.3 s for the healthy eyes and 2.0 ± 0.2 s for the dry eyes) were shorter than the results of the current study. These differences may be explained, in part, by differences in the version of the software by Oculus. The software version was Keratograph 4 in Hong et al.'s study, while in Koh et al.'s and our study, the software was Keratograph 5M.

Using the K5M, TMH was imaged and easily quantified in both the healthy and DED groups. Previous studies [10, 11, 24], using optical coherence tomography, have found it to be significantly decreased in TMH values of dry eyes compared with those of healthy eyes. In the current study, the mean TMH values were 0.22 ± 0.07 mm for DED group and 0.27 ± 0.12 for healthy group. Correspondingly, Hong et al. [16] compared dry eye patients with healthy controls and reported similar lower values for TMH (0.269 ± 0.011 versus 0.379 ± 0.015 mm, resp.) measured by Keratograph 4. Koh et al. [12] also reported that the TMH values were 0.14 ± 0.03 and 0.20 ± 0.05 mm in patients with DED and healthy subjects, respectively. It was shown that our results were somewhere between the results of those two studies. These differences may be explained, in part, by differences in diagnostic criteria for dry eye and in different age stages. Moreover, the poor resolution of TMH images made it difficult to correctly delineate the tear meniscus, especially in the DED group with lower TMH. This might eventually cause the measurement deviation with different examiners.

Age is an important risk factor for DED, and age has been shown to affect the TMH values, with tear menisci in general decreasing with age [24, 25]. The mean ages of the groups in the current study were close to each other, and there was no statistically significant difference between the groups (*P* = 0.403). Therefore, a difference in age is not the reason for the observed differences in the TMH of the groups.

However, the current study had a few limitations. This was an observational cross-sectional study. It is not possible to determine how the longitudinal change of DED progression is related to the TMH and NIKBUTs. The sample size of this study was relatively small and therefore the results should be interpreted cautiously. The intersession repeatability, which is a test in a different day with the same examiner, was not included in this study. Further study with long-term follow-up, larger sample size, and intersession repeatability test is required to explore our findings and the findings of others in greater detail.

In conclusion, noninvasive ocular surface examinations using K5M showed differences in the TMH and NIKBUTs in DED and healthy groups. And K5M may provide a simple, noninvasive screening test for dry eye with acceptable repeatability and reproducibility. It should be considered as an alternative method in the diagnosis and follow-up of patients with DED. Whether its results are more dependable than those obtained with the Schirmer test and FBUT needs further evaluation in studies with a larger patient population.

## Figures and Tables

**Table 1:** Characteristics of the study population.

Parameters	Healthy (*n* = 42)	Dry eye disease (*n* = 42)	*P* value
Age (year)	38.76 ± 13.18	41.43 ± 15.77	0.403
Gender (male/female)	12/30	14/28	0.637
OSDI (score)	3.74 ± 6.90	30.37 ± 15.11	<0.001
FBUT (s)	9.15 ± 3.51	4.59 ± 1.71	<0.001
Schirmer test (mm/5 min)	15.48 ± 8.68	8.21 ± 5.68	<0.001
Oxford scale	0.00 ± 0.00	1.16 ± 1.54	<0.001
Corneal sensation (mm)	6.07 ± 0.09	5.63 ± 0.47	<0.001
TMH (mm)	0.27 ± 0.12	0.22 ± 0.07	0.02
NIKBUT-first (s)	7.36 ± 3.99	5.57 ± 3.31	0.028
NIKBUT-average (s)	10.35 ± 4.22	8.08 ± 4.08	0.014

FBUT = fluorescein tear breakup time; NIKBUT = noninvasive Kertograph tear breakup time; TMH = tear meniscus height; OSDI = Ocular Surface Disease Index.

**Table 2:** Intraexaminer repeatability and interexaminer reproducibility of TMH and NIKBUTs in Healthy and DED groups.

Parameters	Healthy (*n* = 42)						Dry eye disease (*n* = 42)					
	Mean ± SD	Precision	Repeatability	CV%	ICC	95% CI for ICC	Mean ± SD	Precision	Repeatability	CV%	ICC	95% CI for ICC
Intraexaminer repeatability												
TMH (mm)	0.26 ± 0.12	0.10	0.14	18.89	0.84	0.75 to 0.90	0.24 ± 0.08	0.08	0.11	15.96	0.76	0.64 to 0.85
NIKBUT first (s)	7.25 ± 3.45	3.70	5.24	26.10	0.75	0.62 to 0.84	5.77 ± 3.04	2.67	3.76	23.54	0.82	0.73 to 0.89
NIKBUT average (s)	10.61 ± 3.93	3.96	5.60	19.06	0.78	0.66 to 0.86	8.27 ± 3.86	3.04	4.29	18.71	0.85	0.77 to 0.91
Interexaminer reproducibility												
TMH (mm)	0.26 ± 0.15	0.10	0.14	19.76	—	—	0.24 ± 0.08	0.08	0.11	16.08	—	—
NIKBUT first (s)	7.35 ± 3.68	2.92	4.13	20.30	—	—	5.87 ± 3.07	2.51	3.55	21.85	—	—
NIKBUT average (s)	10.71 ± 4.11	3.65	5.15	17.37	—	—	8.21 ± 3.93	2.78	3.94	17.30	—	—

SD = standard deviation; TMH = tear meniscus height; NIKBUT = noninvasive Keratograph tear breakup time; CV = coefficient of variation; ICC = intraclass correlation coefficient; 95% CI = 95% confidence interval for the mean.
